# Three-dimensional radiochromic film dosimetry for volumetric modulated arc therapy using a spiral water phantom

**DOI:** 10.1093/jrr/rrt059

**Published:** 2013-05-17

**Authors:** Masao Tanooka, Hiroshi Doi, Hideharu Miura, Hiroyuki Inoue, Yasue Niwa, Yasuhiro Takada, Masayuki Fujiwara, Toshiyuki Sakai, Kiyoshi Sakamoto, Norihiko Kamikonya, Shozo Hirota

**Affiliations:** 1 Department of Radiology, Hyogo College of Medicine, 1-1, Mukogawa-cho, Nishinomiya City, Hyogo, 663-8501, Japan; 2 Department of Radiological Technology, Hyogo College of Medicine College Hospital, Hyogo, 1-1, Mukogawa-cho, Nishinomiya City, Hyogo, 663-8501, Japan

**Keywords:** spiral water phantom, VMAT, radiochromic film, film dosimetry, QA, spiral phantom

## Abstract

We validated 3D radiochromic film dosimetry for volumetric modulated arc therapy (VMAT) using a newly developed spiral water phantom. The phantom consists of a main body and an insert box, each of which has an acrylic wall thickness of 3 mm and is filled with water. The insert box includes a spiral film box used for dose-distribution measurement, and a film holder for positioning a radiochromic film. The film holder has two parallel walls whose facing inner surfaces are equipped with spiral grooves in a mirrored configuration. The film is inserted into the spiral grooves by its side edges and runs along them to be positioned on a spiral plane. Dose calculation was performed by applying clinical VMAT plans to the spiral water phantom using a commercial Monte Carlo-based treatment-planning system, Monaco, whereas dose was measured by delivering the VMAT beams to the phantom. The calculated dose distributions were resampled on the spiral plane, and the dose distributions recorded on the film were scanned. Comparisons between the calculated and measured dose distributions yielded an average gamma-index pass rate of 87.0% (range, 91.2–84.6%) in nine prostate VMAT plans under 3 mm/3% criteria with a dose-calculation grid size of 2 mm. The pass rates were increased beyond 90% (average, 91.1%; range, 90.1–92.0%) when the dose-calculation grid size was decreased to 1 mm. We have confirmed that 3D radiochromic film dosimetry using the spiral water phantom is a simple and cost-effective approach to VMAT dose verification.

## INTRODUCTION

Volumetric modulated arc therapy (VMAT) is a dynamic treatment technique, in which gantry speeds, dose rates, and positions of the multi-leaf collimator (MLC) and jaws are simultaneously varied during gantry rotation [1–3]. However, dose discrepancy during VMAT delivery has been reported due to limitations in hardware performance of the treatment unit [4–6]. It is therefore recommended that each VMAT facility establish an appropriate VMAT quality assurance (QA) procedure. It is also suggested that dose verification be performed in an entire volume covering all beam cross sections during gantry rotation. A conventional method has been the use of radiographic films sandwiched between slab phantoms, in which the films are oriented either parallel or perpendicular to the beam.

Recently, various QA devices for VMAT delivery verification have been developed [7–12], and film dosimetry for dose verification on cylindrical planes has also been reported [13]. Paliwal *et al*. have proposed a spiral solid phantom for intensity-modulated radiation therapy (IMRT) and tomotherapy [14, 15], in which the spiral solid phantom is specified as a water-equivalent solid cylinder with a narrow spiral cavity, into which is inserted a radiographic film. In this configuration, there is a narrow air gap between the spiral cavity wall and the inserted film, possibly causing position instability of the film and thus dose discrepancy between calculation and measurement.

Radiation dosimetry is performed based on the absorbed dose to water. A radiochromic film can be used in water, which solves a dose-perturbation problem caused by the air gap between the film and the solid phantom. Previous film dosimetry using the radiochromic film was limited to 2D planar dosimetry. We have performed 3D radiochromic film dosimetry for VMAT dose verification using a new spiral water phantom.

## MATERIALS AND METHODS

**Fig. 1.**
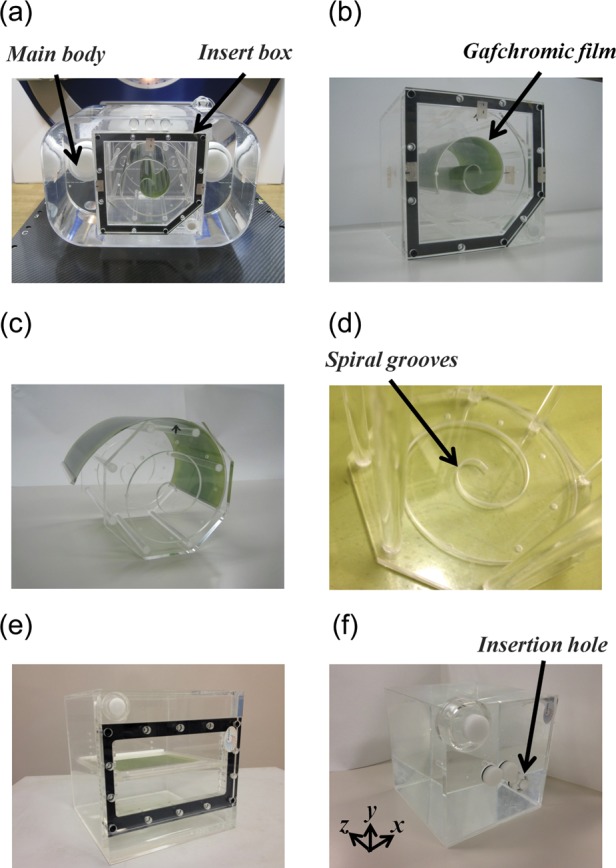
Photographs of the spiral water phantom made of acrylic resin. (**a**) The spiral water phantom consists of a main body and an insert box, each with a wall thickness of 3 mm and filled with water. (**b**) A spiral film box, which is an insert box for dose-distribution measurement, includes a film holder for positioning an EBT2 Gafchromic film. (**c, d**) The film holder has two parallel walls (10-mm thickness) at a distance of 130 mm from each other. The side edges of the film (130 mm × 254 mm) are inserted into mirrored spiral grooves (0.5 mm width, 5 mm depth) provided on the facing inner surfaces of the parallel walls, thereby allowing the film to be positioned on a spiral plane with an arc length of 254 mm. After placing the film in the film holder, the film holder was moved into the spiral film box and the spiral film box was placed inside the main body. (**e**) A film box is another insert box for dose calibration. (**f**) An ionization chamber box is still another insert box for point dose measurement.

**Fig. 2.**
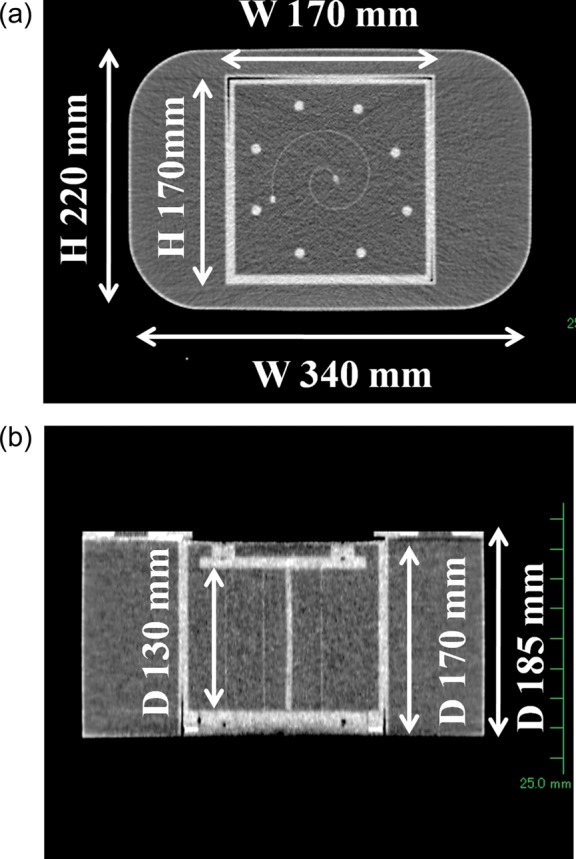
CT images of the spiral water phantom when the spiral film box was placed inside the main body. (**a**) Transverse view of the phantom. (**b**) Coronal view of the phantom.

Figure 1 shows the spiral water phantom, which is made of acrylic resin (R-tech. Inc., Tokyo, Japan). The phantom consists of a main body and an insert box, each of which has a wall thickness of 3 mm and is filled with water. The insert box includes a spiral film box used for dose distribution measurement, and a film holder for positioning a radiochromic film. An EBT2 Gafchromic film (International Specialty Products, New Jersey, USA) with an original size of 8 × 10 inches was cut to a size of 130 mm × 254 mm. The film holder has two parallel walls (10-mm thickness) at a distance of 130 mm from each other. The film was inserted along its sides into mirrored spiral grooves (0.5 mm width, 5 mm depth) on the facing surfaces of the parallel walls, thereby allowing the film to be positioned on a spiral plane with an arc length of 254 mm. After placing a film in the film holder, the film holder was moved into the spiral film box and the spiral film box was placed inside the main body. The insert box also contains a film box for dose calibration (Fig. 1e), and an ionization chamber box for point dose measurement (Fig. 1f). These insert boxes are also made of acrylic resin which have a wall thickness of 3 mm and are filled with water. The ionization chamber box has an insertion hole, and the dose at a specific point of (*x, y, z*) = (2, − 2, 0) cm can be measured both by the film and the ionization chamber for comparison. Figure 2 shows CT images of the spiral water phantom with the spiral film box placed inside the main body.

**Fig. 3.**
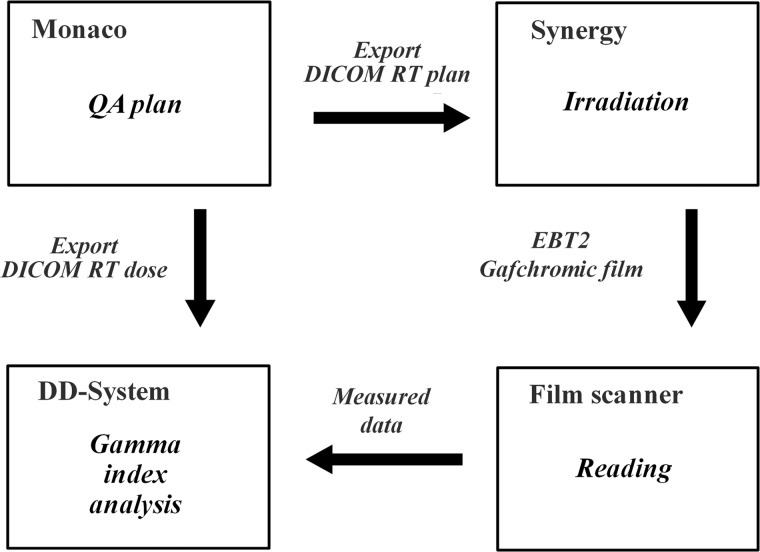
A workflow of 3D radiochromic film dosimetry for VMAT using the spiral water phantom. A clinical VMAT plan was applied to the phantom to calculate dose distributions. The VMAT plan was also exported to a linac controller for dose delivery. The calculated dose distributions were resampled on the spiral plane for dose verification using the gamma index.

Figure 3 depicts a workflow of 3D radiochromic film dosimetry for VMAT using the spiral water phantom. A VMAT plan was created using a commercial Monte Carlo-based treatment-planning system (TPS), Monaco 2.03 (Elekta, Missouri, USA), and applied to the spiral water phantom with a dose-calculation grid size of 2 mm or 1 mm and a variance of 2%. Dose measurement was performed by delivering the VMAT beams to the spiral water phantom using an Elekta Synergy linear accelerator (Elekta, Crawley, UK) equipped with an MLC with a leaf width of 1 cm. After dose delivery, the EBT2 Gafchromic film was carefully taken out of the spiral water phantom, carefully wiped to remove water drops, then hung up to dry. After 24 h, which is regarded as the proper time for optical density growth to reach a plateau [13, 16], the EBT2 Gafchromic film was scanned by a flatbed scanner GT-X970 (SEIKO EPSON, Nagano, Japan) in landscape scan mode with 48-bit color at a resolution of 72 dpi and gamma corrections turned off, and the response in the red color channel was extracted and used for the calibration and for dose measurement. Built-in scanner software, Film-Scan-New (version 4.0), was used with a film analysis device, DD-System (R-tech. Inc., Tokyo, Japan). All EBT2 Gafchromic films came from the same film batch and were processed under the same conditions.

In this study, a dose-response curve for dose calibration was obtained for 10-MV photon beams. The EBT2 Gafchromic film was cut into nine pieces of 7 × 12.5 cm^2^ each. After each film piece was placed in the film box for dose calibration, the film box was further placed inside the main body, with the film plane perpendicular to the central axis of a vertical beam with a gantry angle of 0°. The source to film distance was 100 cm and the field size was 10 × 10 cm^2^. The following doses were delivered to the calibration film: 0, 2.4, 4.0, 8.0, 19.9, 39.8, 79.6, 159.2, 238.8 and 318.3 cGy, which corresponded to monitor units ranging from 0 to 400. After 24 h, all film pieces were scanned with the flatbed scanner and the Film-Scan-New software.

The calculated dose distribution was saved as a DICOM RT dose file in the TPS and transferred to a film analysis device, the DD-System, in which the calculated dose distribution was resampled using DD-IMRT (version 9.4) on the spiral plane for dose verification using the gamma index. Other TPSs can also be employed provided that they can export a DICOM RT dose file.

Archimedes' spiral [14] was employed with the spiral trajectory expressed in polar coordinates as follows:

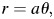

where *r* was the radial distance, θ was the polar angle (θ ≥ *π/3*), and the constant *a* was set at 0.5129.

The minimum polar angle of *π/3* was due to the limited mechanical flexibility of the film. The constant factor of 0.5129 was chosen so that the spiral plane includes Cartesian coordinates of (*x*, *y, z*) = (2, − 2, 0) cm, at which location an ionization chamber insertion hole was also provided for point dose measurement.

Initially, dose measurement was performed by delivering a single field beam (6 × 6 cm^2^) to the spiral water phantom at a 0° gantry angle with a photon energy of 10 MV. Nine prostate VMAT plans with a photon energy of 10 MV were then randomly selected for the present study. A dose of 74 Gy in 37 fractions was prescribed for 95% of the planning target volume (PTV). Three different dose comparisons were made between the measurement and the calculation, in which the dose-calculation grid size was set to 2 mm. First, the gamma index was calculated with a distance to agreement of 3 mm and a dose difference of 3% relative to each measured dose, under a dose threshold of 50% of the maximum dose on each plane. Second, the isodose contours of the measurement and calculation were overlaid. Third, the calculated and measured radial dose profiles were overlaid at 12° intervals with the origin at the film center.

In addition, the pass rates of the gamma-index analysis were compared on the spiral plane and on central orthogonal planes for the nine prostate VMAT plans. Lastly, the dose distributions were recalculated with a dose-calculation grid size of 1 mm, and the impact of the dose-calculation grid size on the pass rates was analyzed using the Wilcoxon signed-rank test with a statistical significance level of 5%.

## RESULTS

**Fig. 4.**
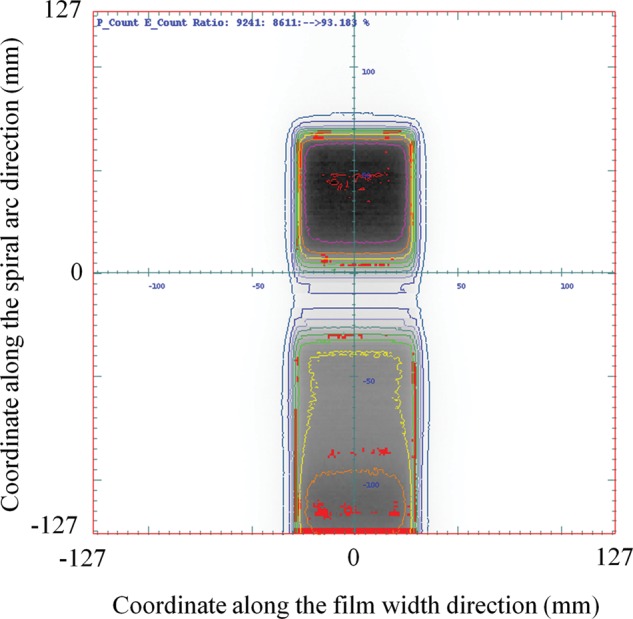
A gamma distribution on the spiral plane for a single field plan. Dose measurement was performed by delivering a single field beam (6 × 6 cm^2^) to the spiral water phantom at 0° gantry angle with a photon energy of 10 MV. The red areas have gamma indices larger than one under the criteria of a dose difference of 3% relative to each measured dose and a distance to agreement of 3 mm. A pass rate of 93.2% was obtained with a dose-calculation grid size of 2 mm for this plan.

Figure 4 shows the gamma distribution on the spiral plane for the single field plan. The red areas have gamma indices > 1 under the criteria of a dose difference of 3% relative to each measured dose and a distance to agreement of 3 mm. A pass rate of 93.2% was obtained with a dose-calculation grid size of 2 mm for this plan.

**Fig. 5.**
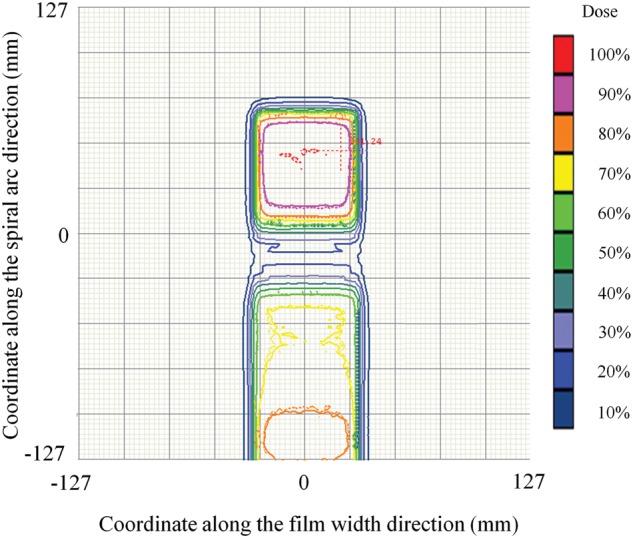
A comparison of isodose contours between calculated (solid line) and measured (dotted line) dose distributions with an isocontour step size of 10%, for the single field plan shown in Fig. 4.

Figure 5 shows a comparison of isodose contours between the calculated and measured dose distributions with an isocontour step size of 10%, for the single field plan shown in Fig. 4.

**Fig. 6.**
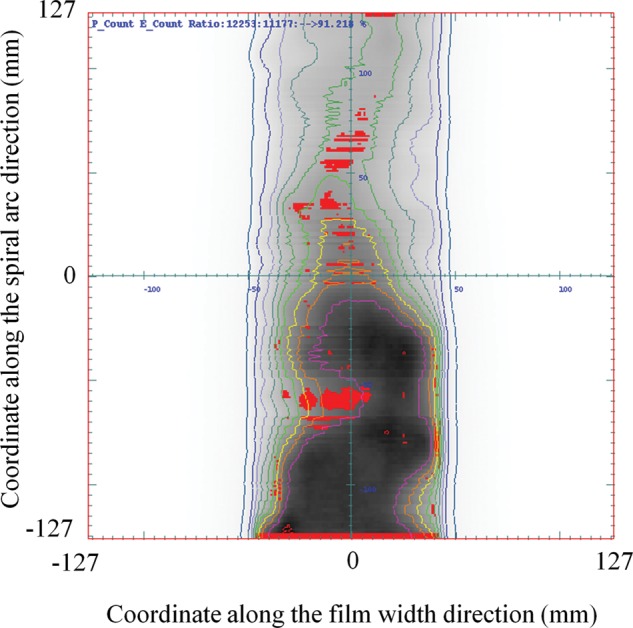
A gamma distribution on the spiral plane for a typical prostate VMAT plan. The red areas have gamma indices larger than one under the criteria of a dose difference of 3% relative to each measured dose and a distance to agreement of 3 mm. A pass rate of 91.2% was obtained with a dose-calculation grid size of 2 mm for this plan.

Figure 6 shows the gamma distribution on the spiral plane for a typical prostate VMAT plan. A pass rate of 91.2% was obtained with a dose-calculation grid size of 2 mm for this plan. Good agreement was observed with the single field plan.

**Fig. 7.**
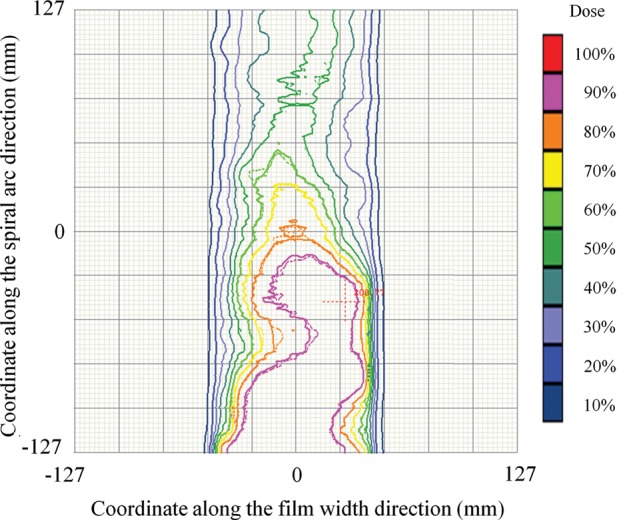
A comparison of isodose contours between calculated (solid line) and measured (dotted line) dose distributions with an isocontour step size of 10%, for the VMAT plan shown in Fig. 6.

Figure 7 shows a comparison of isodose contours between the calculated and measured dose distributions with an isocontour step size of 10%, for the VMAT plan shown in Fig. 6. Again, good agreement was observed with the single field plan.

**Fig. 8.**
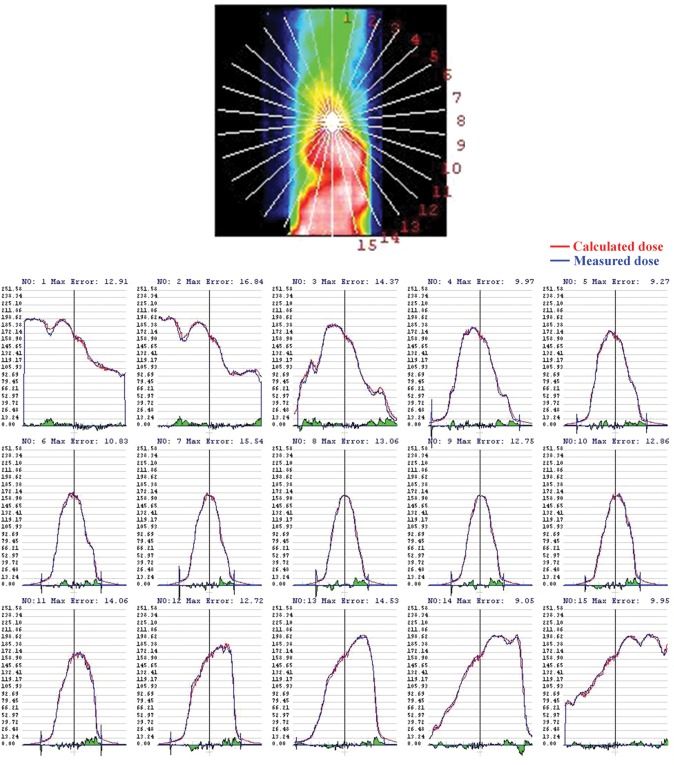
Comparisons between calculated (red line) and measured (blue line) radial dose profiles at 12° intervals for the VMAT plan with the origin at the film center. Each of the two dose profiles was normalized to each maximum dose, and showed good agreement.

Figure 8 demonstrates comparisons between the calculated and measured radial dose profiles at 12° intervals for the VMAT plan with the origin at the film center. Each of the two dose profiles was normalized to each maximum dose, and showed good agreement.

Table 1 shows comparisons of gamma-index pass rates (3 mm/3% criteria) on a spiral plane and on central orthogonal planes for nine prostate VMAT plans. The pass rate was calculated with a dose threshold of 50% of the maximum dose to each plane. The pass rates on the spiral plane were significantly less than those on the central orthogonal planes. The pass rate difference between the spiral plane and each of the central orthogonal planes was statistically significant (*P* = 0.011).

**Table 1.** Comparison of gamma-index pass rates (3 mm/3% criteria) on a spiral plane and on central orthogonal planes for nine prostate VMAT plans with a dose-calculation grid size of 2 mm

Plane	Mean	Maximum	Minimum	SD	Plane	Mean	Maximum	Minimum	SD
					Transverse	95.9%	99.9%	92.4%	2.4%
Spiral	87.0%	91.2%	84.6%	2.4%	Coronal	96.5%	98.1%	93.1%	2.0%
					Sagittal	95.9%	99.0%	93.1%	1.7%

The pass rate was calculated with a dose threshold of 50% of the maximum dose on each plane. The pass rate difference between the spiral plane and each of the central orthogonal planes was statistically significant (*P* = 0.011). SD = standard deviation. VMAT plans, *n* = 9.

**Fig. 9.**
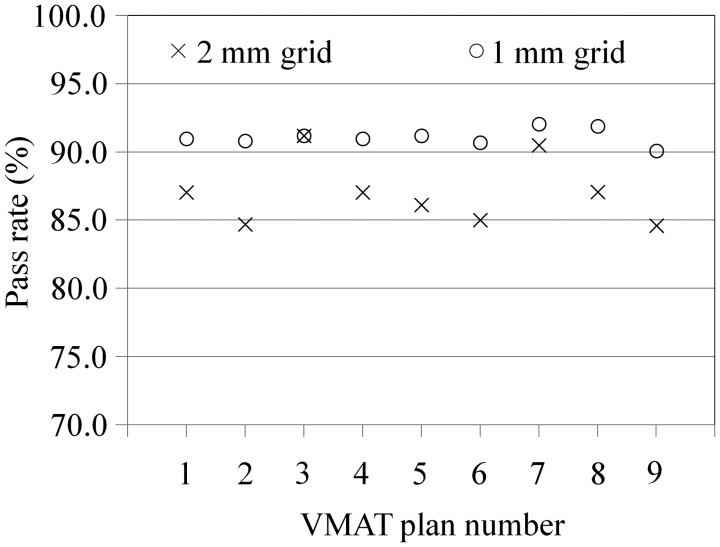
Comparisons of the gamma-index pass rates with dose-calculation grid sizes of 1 mm (circle) and 2 mm (cross) for the nine VMAT plans. The pass rates were increased beyond 90% (average, 91.1%; range, 90.1–92.0%) when the calculation grid size was decreased to 1 mm. The pass rate difference between the different dose-calculation grid sizes was statistically significant (*P* = 0.011).

Figure 9 shows comparisons of the gamma-index pass rates with dose-calculation grid sizes of 1 mm and 2 mm for the nine VMAT plans. The pass rates were increased beyond 90% (average, 91.1%; range, 90.1–92.0%) when the calculation grid size was decreased to 1 mm. The pass rate difference between the different dose-calculation grid sizes was statistically significant (*P* = 0.011).

The dose calculation required approximately 2 h for the 2-mm grid, and 16 h for the 1-mm grid using an HP xw8600 workstation under the same variance condition of 2%.

## DISCUSSION

We evaluated 3D radiochromic film dosimetry for VMAT delivery verification using a newly developed spiral water phantom with radiochromic film. The calculated and measured dose distributions were qualitatively in good agreement (Figs 6–8, Table 1). However, these results were dependent on the dose-calculation grid size employed by the TPS (Fig. 9). In other words, the pass rates of the gamma index increased when a dose-calculation grid size of 1 mm was employed. This means that the film position was sufficiently accurate, and the dose-calculation accuracy in the TPS increased when the calculation grid size decreased to 1 mm. Chung *et al*. investigated dose uncertainty as a function of the dose-calculation grid size and found that the relative dose uncertainty increased when the calculation grid size increased [17]. Our results showed a similar tendency, and a calculation grid size of 1 mm was deemed preferable. However, the Monte Carlo computation time with a dose-calculation grid size of 1 mm was prohibitively large and not clinically acceptable with our current workstation.

According to ESTRO Booklet No. 9 [18], the dose-map evaluation should include both high and low dose regions in the plane. 3D radiochromic film dosimetry using the spiral water phantom meets this requirement. However, when the dose threshold of 50% was lowered for the evaluation of low-dose regions, the pass rates of our gamma-index calculation greatly decreased. This was because the percent dose difference employed in our gamma-index formula was normalized to each locally measured dose with a dose threshold of 50% of the maximum dose to each plane. Thus, the gamma index depends on the normalization procedure and it is desirable to employ global dose normalization criteria for low-dose regions. In other words, to evaluate the low-dose region with doses less than 50% of the prescribed dose, the gamma index needs to be calculated using VanDyk dose normalization [19], in which 100% dose is defined by a constant reference dose such as a prescribed dose or maximum dose to a plane. Meanwhile, we evaluated low-dose regions by other methods such as dose profiles or isodose contours.

In 3D radiochromic film dosimetry using the spiral water phantom, conventional 2D gamma-index analysis may not be appropriate in high-dose gradient regions due to phantom set-up uncertainty regarding the direction perpendicular to the film surface. A 3D gamma-index analysis may solve this problem [20].

The pattern of characteristic horizontal stripes in the irradiated region was observed in Figs 4 and 6. Because the errors appeared at specific points on a spiral trajectory, they were considered an influence of dose deviations caused by dose interpolation and the 2D gamma method described above.

An advantage of the current spiral water phantom is that there is no air gap between the phantom material and the EBT2 Gafchromic film, thereby preventing dose measurement instability. In addition, compared to a radiographic film, a radiochromic film has a relatively small beam-angle dependence of sensitivity, as well as a small energy-dependence [16, 18]. For this reason, we assume that the difference of the geometric conditions such as the beam angles, field sizes, and measurement depths between the dose calibration and the dose measurement may be insignificant. Furthermore, the dose response of the EBT2 Gafchromic film was very similar to that of the ion chamber scanned in a water phantom [21, 22]. The fine resolution of the film dosimetry enables the accurate and detailed analysis of the gamma index, and this film system may accordingly continue to be the most practical and cost-effective device, with a much higher spatial resolution than any other array-based dosimetry systems.

## CONCLUSION

We have validated the use of 3D radiochromic film dosimetry using a spiral water phantom with satisfactory results and confirmed that the system is a simple and cost-effective approach to VMAT dose verification.

## FUNDING

This work was partially supported by a Grant-in-Aid for University Reform 2010 (Cancer Professional Training Plan) from the Ministry of Education, Culture, Sports, Science and Technology, Japan.
